# Survival outcome among patients with Ewing’s sarcoma of bones and joints: a population-based cohort study

**DOI:** 10.1590/1516-3180.2017.0236230917

**Published:** 2017-12-04

**Authors:** Zi-Hao Wan, Zhi-Hao Huang, Liao-Bin Chen

**Affiliations:** I MD, MSc. Surgeon, Department of Orthopedic Surgery, Zhongnan Hospital, Wuhan University, Wuhan, Hubei Province, China.; II MD, MSc. Surgeon, Department of Colorectal and Anal Surgery, Zhongnan Hospital, Wuhan University, Wuhan, Hubei Province, China.; III MD. Surgeon, Department of Orthopedic Surgery, Zhongnan Hospital, Wuhan University, Wuhan, Hubei Province, China.

**Keywords:** Bone and bones, Sarcoma, Ewing, Survival analysis, Retrospective studies

## Abstract

**BACKGROUND:**

The aim here was to elucidate the current survival condition of patients diagnosed with Ewing’s sarcoma of the bones and joints and determine independent risk factors associated with the prognosis.

**DESIGN AND SETTING:**

Retrospective cohort study based on the Surveillance, Epidemiology and End Results (SEER) database in the United States.

**METHODS:**

We identified 397 patients who were diagnosed with Ewing’s sarcoma of the bones and joints between January 2004 and December 2013. The multivariate Cox proportional hazards model was used to determine factors associated with the risk of death by adjusting for various factors.

**RESULTS:**

The one, two and five-year disease-specific survival rates were 89.08%, 78.08% and 62.47%, respectively. The factors related to death were age (≥ 18 years versus < 18 years; hazard ratio, HR = 1.77; 95% confidence interval, CI: 1.38-2.31); tumor site (extremity versus spine and pelvis; HR = 2.03; 95% CI: 1.31-2.62); tumor size (> 10 cm versus ≤ 10 cm; HR = 1.78; 95% CI: 1.34-2.56); and type of treatment (surgery alone versus radiotherapy with surgery; HR = 0.51; 95% CI: 0.38-0.89; or radiotherapy alone versus radiotherapy with surgery; HR = 1.61; 95% CI: 1.10-2.39; or no treatment versus radiotherapy with surgery; HR = 1.86; 95% CI: 1.23, 2.58).

**CONCLUSIONS:**

Patients with Ewing’s sarcoma showed poor survival in situations of age ≥ 18 years, tumor size > 10 cm, receiving radiotherapy alone and receiving no treatment. Patients undergoing surgery alone had better survival.

## INTRODUCTION

Ewing’s sarcoma is a rare cancer that accounts for less than 10% of all malignancies existing in the human body. It stems from primitive neuroepithelial cells, which are able to differentiate into various mesenchymal cells, and has a propensity to metastasize to distant sites at an early stage . This cancer typically occurs in adolescents and young adults, accompanied by a very poor prognosis. It is considered to be a high-grade malignancy, ranking second in the list of malignant bone tumors.^1^^,^^2^^,^^3^^,^^4^^,^^5^^,^^6^^,^^7^ It is commonly considered to be an extremely aggressive osteolytic cancer that usually occurs in the bones of the limbs and pelvis and it can metastasize to distant locations such as bone marrow, the lungs and other soft tissues at an early stage.^1^^,^^3^^,^^7^

In the United States, the overall incidence rate of Ewing’s sarcoma is approximately 0.1 case per 100,000 individuals per year, and this rate had not undergone any obvious change over past 30 years. An estimated 90% of these patients are under 20 years old, and the death rate is approximately 0.05 cases per 100,000 individuals per year. Additionally, most cases of Ewing’s sarcoma of the bones and joints are found in the limbs, pelvis or spine.^4^^,^^8^

Nonetheless, there is a lack of survival studies on Ewing’s sarcoma arising in the bones and joints and associated prognostic factors, based on up-to-date data on nationwide populations.

## OBJECTIVE

The purpose of this study was to demonstrate the survival conditions of patients with Ewing’s sarcoma of the bones and joints and determine independent risk factors associated with their prognosis.

## METHODS

The Surveillance, Epidemiology and End Results (SEER) database named “Incidence-SEER 18 Regs Research Data + Hurricane Katrina Impacted Louisiana Cases, Nov 2015 Sub (1973-2013 varying)” was selected to perform a population-based search for patients suffering from Ewing’s sarcoma of the bones and joints between January 2004 and December 2013.

The SEER Program^9^ is supported by the National Cancer Institute of the United States and has provided statistical information on tumor cases since 1973. It collects data on cases diagnosed with cancer throughout the United States, with an estimated 28% of the United States population covered. The SEER registry is a validated database that is frequently applied for cancer survival studies. The National Cancer Institute does not require institutional review board approval for SEER studies because it is an unidentified public-use database.

Histological International Classification of Diseases (ICD) codes (ICD-0-3) were used to identify Ewing’s sarcoma (9260/3), (9364/3). Site-specific codes (C40.0-C40.3, C40.8, C40.9, C41.2, C41.4 and C41.8) were used to screen for tumors originating in the extremities, pelvis and spine, while the codes for bones of the skull and face, mandible, rib, sternum, clavicle and associated joints were not included.

**Figure 1:**
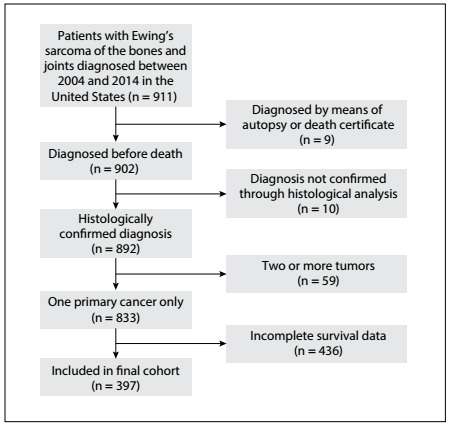
Flow chart for selection of the study cohort.

The following primary data were drawn from the database for analysis: age at diagnosis, sex, race, tumor site, tumor size, tumor grade, type of treatment, cause of death and survival in months. Patients diagnosed through either autopsy or the death certificate were excluded. Those who presented secondary malignancies at the time of diagnosis or whose diagnoses were not confirmed by means of histopathological evaluation were also excluded. Cases without complete information were excluded. The inclusion and exclusion procedure is showed in the flow chart of Figure 1.

Well-differentiated and moderately differentiated histological features were classified as low grade while poorly-differentiated and undifferentiated histological types were classified as high grade. The pathological tumor-node-metastasis (pTNM) classification was used in cases of patients who underwent tumor resection, because their tumor gradings could be confirmed through analysis under a microscope. Because the sixth edition of the staging system of the American Joint Commission on Cancer was used in the SEER database starting in 2004 and the most recent update for this database was in 2013 (which was when the present study began), we chose to use this period (2004 to 2013) for the present study.

The main result from this study was the disease-specific survival (DSS). Descriptive statistics were calculated for all factors. The DSS was calculated by means of the Kaplan-Meier method, and the log-rank test was applied to appraise the deviations. We calculated hazard ratios (HRs) and the 95% confidence interval (CI) in the Cox proportional hazards model after adjusting for various variables. The SEER*Stat application version 8.3.2 (IMS Inc., USA) was used to extract primary data. All statistical analyses was finished in the SPSS software, version 23 (IBM Corp., USA). Differences between groups were taken to be statistically significant when the P value was less than 0.05.

## RESULTS

During the 10-year period, 397 patients diagnosed with Ewing’s sarcoma of the bones and joints were included (249 males and 148 females). Table 1 shows the distribution of the patients’ characteristics in the study. The mean age (with SD) at diagnosis was 18.5 (12.4) years. 65.1% of the patients were aged < 18 years. Tumors arising from the extremities accounted for 55.1%. 378 tumors (95.2%) were histologically confirmed to be poorly-differentiated or undifferentiated tumors. The mean tumor size at the time of diagnosis was 10.3 cm (5.3). 86 patients (21.6%) received surgery with radiotherapy, 139 (35.1%) underwent surgical resection alone, 102 (25.7%) underwent radiotherapy alone and 70 (17.6%) received no treatment.

**Table 1:** Characteristics of patients with Ewing’s sarcoma of the bones and joints

Characteristic	Total	
	N	%
Patients	397	100
Sex		
Female	148	37.2
Male	249	62.8
Age, years		
< 18	251	63.2
≥ 18	146	36.8
Mean (with standard deviation)	18.5 (12.4)	-
Median	17.8	-
Race		
White	351	88.4
Black	15	3.8
Other	31	7.8
Tumor site		
Extremity	219	55.1
Spine and pelvis	178	44.9
Tumor grade		
Low	19	4.8
High	378	95.2
Tumor size		
≤ 10	173	43.6
> 10	224	56.4
Mean (with standard deviation)	10.3 (5.3)	-
Median	9.6	-
Treatment		
Surgery with radiotherapy	86	21.6
Surgery alone	139	35.1
Radiotherapy alone	102	25.7
None	70	17.6

**Figure 2:**
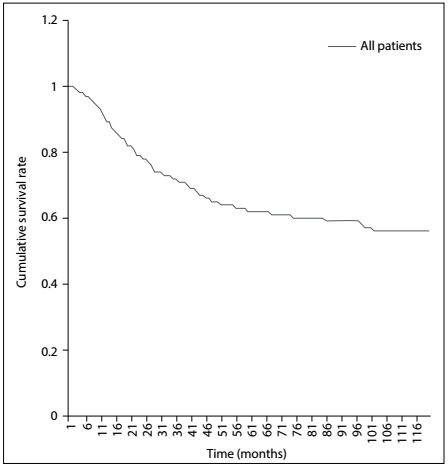
Kaplan-Meier survival curve for all patients.

**Figure 3:**
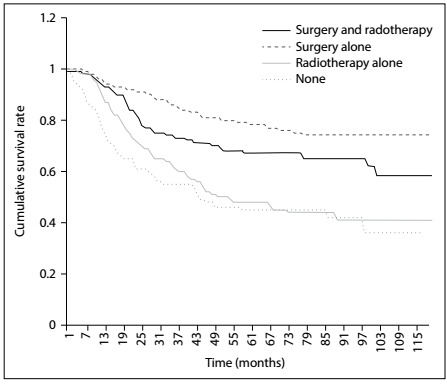
Kaplan-Meier survival curves for all patients, stratified according to type of treatment.

**Figure 4:**
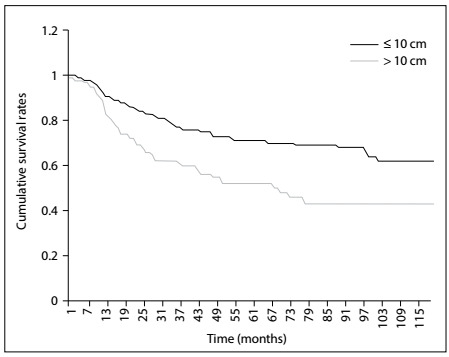
Kaplan-Meier survival curves for all patients, stratified according to tumor size.

**Figure 5:**
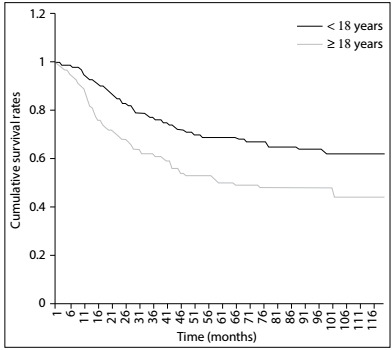
Kaplan-Meier survival curves for all patients, stratified according to age.

The overall one, two and five-year survival rates after diagnosis were 89.08%, 78.08% and 62.47%, respectively (Figure 2). The five-year relative survival rates were 78.4%, 66.9%, 47.8% and 44.8% for patients receiving surgery, surgery with radiotherapy, radiotherapy alone and no therapy, respectively (Figure 3). Overall, patients with tumor size ≤ 10 cm had a higher five-year survival rate than did those with tumor size > 10 cm (70.8% versus 52.4%; P < 0.001) (Figure 4). The five-year survival rate were 68.7% and 50.2% for those < 18 and ≥ 18 years (Figure 5).

Table 2 shows the results from univariate and multivariate Cox proportional hazards analyses. Age ≥ 18 years (HR = 1.77; 95% CI = 1.33-2.01), tumor originating in the spine and pelvis (HR = 2.03; 95% CI = 1.31-2.62), tumor size > 10 cm (HR = 1.78; 95% CI = 1.24-2.35), radiotherapy alone (HR = 1.61; 95% CI = 1.10-2.39) and no treatment (HR = 1.86; 95% CI = 1.23-2.58) were associated with increased risk of mortality, while receiving surgery alone (HR = 0.51; 95% CI = 0.38-0.89) was an independent predictor for longer survival.

**Table 2:** Cox model with hazard ratios and 95% confidence intervals for mortality associated with covariates, among patients with Ewing’s sarcoma of bones and joints

Variable	Crude			Adjusted*		
	HR	(95% CI)	P-value	HR	(95% CI)	P-value
Sex						
Female	1	(reference)		1	(reference)	
Male	1,47	(1.12-1.93)	< 0.05	1.19	(0.98-1.71)	0.074
Age						
< 18 years	1	(reference)		1	(reference)	
≥ 18 years	1,89	(1.47-2.44)	< 0.05	1.77	(1.38-2.31)	< 0.05
Race						
White	1	(reference)		1	(reference)	
Black	1.68	(1.27-2.07)	< 0.05	1.52	(1.37-1.86)	0.075
Other	0.63	(0.49-1.53)	0.38	0.91	(0.68-1.74)	0.84
Tumor site						
Extremity	1	(reference)		1	(reference)	
Spine and pelvis	2.57	(1.74-2.79)	< 0.05	2.03	(1.31-2.62)	< 0.05
Tumor grade						
Low	1	(reference)		1	(reference)	
High	2.37	(0.77-4.29)	0.81	-	-	-
Tumor size						
≤ 10 cm	1	(reference)		1	(reference)	
> 10 cm	2.01	(1.46-2.77)	< 0.05	1.78	(1.34-2.56)	< 0.05
Treatment						
Radiotherapy with surgery	1	(reference)		1	(reference)	
Surgery alone	0.61	(0.42-0.92)	< 0.05	0.51	(0.38-0.89)	< 0.05
Radiotherapy alone	1.66	(1.24-2.51)	< 0.05	1.61	(1.10-2.39)	< 0.05
None	2.27	(1.53-3.38)	< 0.05	1.86	(1.23-2.58)	< 0.05

HR = hazard ratio; CI = confidence interval. *Adjusted for sex, age, race, tumor site, tumor size and treatment.

## DISCUSSION

In our study, we found that the one, two and five-year DSS rates were 89.08%, 78.08% and 62.47%, respectively. These proportions were reported to be higher in a previous investigation.^10^ This difference may reflect that the prognosis of Ewing’s sarcoma originating bones and joints is much worse than that of Ewing’s sarcoma of other parts of human body. Early screening and diagnosis seem to be more important among people at high risk of having Ewing’s sarcoma of bones and joints.

The development of diagnostic methods based on molecular techniques has had a great effect, because typical chromosomal translocations are commonly detected in Ewing’s sarcoma tissue. The reverse transcription-polymerase chain reaction (RT-PCR) and fluorescent in-situ hybridization (FISH) are the most frequent measures applied in fusion gene analysis.

It has been reported that the t(11;22) (q24;q12) translocation is found in 85% of cases of Ewing’s sarcoma.^11^^,^^12^ Yang et al.^13^ further ascertained that FISH and RT-PCR could be applied as reliable molecular diagnostic approaches in cases of Ewing’s sarcoma, and that FISH displayed features of greater sensitivity and stability. Furthermore, in a meta-analysis on 1,412 cases, Li et al.^14^ declared that high levels of serum lactate dehydrogenase (LDH) presaged lower DSS among patients with Ewing’s sarcoma. However, information on these specific molecular indicators is not documented in the SEER database.

The influence of age on survival has always been a matter of debate, with contrary outcomes reported from different studies. In our study, most of the patients included were young, i.e. under 18 years of age, and the median age of our cohort was 17.8 years at the time of diagnosis. This was almost identical to the results reported previously in the worldwide literature, i.e. that most cases of Ewing’s sarcoma not surprisingly emerged before the second decade of life, and that younger patients were likely to have a better prognosis.^15^^,^^16^^,^^17^ Regarding the reasons for this phenomenon, Lee et al.^18^ and Grevener et al.^16^ found that fewer cases among adult patients were treated with chemotherapy. Moreover, elderly patients were more likely to have several comorbidities, including diabetes mellitus hypertension or secondary cancers, which made the situation much more complex.

Tumor size was considered to be a prognostic indicator in our study. We found that the mean size was 10.3 cm, which was almost consistent with the results declared in previous studies. We noticed that sizes larger than 10 cm were associated with a negative impact on DSS. However, there is no consensus regarding any critical cutoff size that might indicate a completely different prognosis for this disease.^19^^,^^20^^,^^21^^,^^22^^,^^23^ In a study on 182 patients, Fizazi et al.^24^ found that tumor size greater than 10 cm was an independent prognostic factor for survival. Canter^25^ also recommended that patients with tumors larger than 10 cm should accept neoadjuvant chemotherapy and investigational therapies, because they were at a high risk of relapse and disease-specific death.

Even so, several studies have asserted that 8 cm might be a more appropriate boundary value. In a retrospective analysis on 220 patients at St. Jude Children’s Research Hospital, Rodriguez-Galindo et al.^23^ found that neoplasm size larger than 8 cm affected survival adversely.

In our analysis, tumors arising from the spine and pelvis were an independent factor for poorer survival. The proportion of the patients who accepted surgery was 56.0%, while 47.5% received radiotherapy. We found that surgery alone, radiotherapy alone, and no treatment were independent risk factors.

There are several explanations for this phenomenon. Oberlin et al.^26^ asserted that it was recommendable that smaller and more peripheral tumors should be dealt with through surgical resection, while larger and more central unresectable entities should be managed with radiotherapy. On the other hand, Granowetter et al.^27^ pointed out that radiotherapy was not appropriate for patients in whom there was no proof of microscopic remainders of malignant tissue after they underwent operations.

Normally, it is accepted that surgery will provide a decisive partial cure. Only when the neoplasm is unresectable or after palliative surgery should radiation therapy be considered. Such patients’ prognoses have been found to be relatively much worse than those of patients who underwent surgery alone.^10^^,^^28^

In a retrospective study on 512 cases, Bacci et al.^29^ concluded that surgical resection is more ideal than radiation therapy for patients with Ewing’s sarcoma of the extremities for whom adequate surgical margins can be achieved. In cases of insufficient surgical margins, high-dose radiotherapy is recommended, while reduced-dose radiotherapy is ineffective.

Furthermore, the main population affected by Ewing’s sarcoma of the bones and joints consists of young people, mostly in their teenage years. For these individuals, excessive exposure to radiation may result in retardation of the development of bones and other organs. This may have side effects of greater severity than those of surgery, which may produce less morbidity. Although surgery is the practice most often used for local control, there are very few randomized controlled trials directly comparing the effects of surgery with those of radiotherapy, and the relative positions of these techniques remain contentious.^28^

In addition, race, sex and tumor grade were not independent factors after adjusting for different variables in our Cox multivariate regression model. On the other hand, these valuables were reported to be independent risk factors in relation to other bone cancers in some previous studies.^19^^,^^20^^,^^22^^,^^30^^,^^31^ Further study regarding whether these factors should be considered as independent risk indicators for the prognosis of patients with Ewing’s sarcoma of the bones and joints is needed.

Our analysis was based on the data documented in the SEER database, which means that we need to acknowledge that there were some limitations relating to our study. Firstly, some variables including data on comorbidities, surgical margins, extent of surgical resection, tumor recrudescence and use of targeted therapy in managing this cancer were missing or not recorded in the database. Secondly, because of the principle of anonymity in the SEER Program, it was impossible for us to contact the patients in order to gain additional information. Thirdly, it also should not be ignored that because of the existence of confounders, the consequences deduced from a retrospective analysis would normally be of lower methodological grade than those from randomized controlled trials. Finally, we were unable to evaluate some specific molecular indicators, such as Ewing’s Sarcoma-Friend leukemia integration 1 transcription factor (EWS-FLI1) and serum LDH, which help in making an early diagnosis and in judging the prognosis.

In spite of these limitations, use of the SEER Program database has significant advantages, in that it provides possibilities for conducting studies of this nature based on large populations suffering from rare types of cancer.

## CONCLUSION

In conclusion, the contemporary five-year DSS rate of Ewing’s sarcoma of the bones and joints was 62.47%. Age ≥ 18 years, tumors originating in the spine and pelvis, tumor size > 10 cm, receiving radiotherapy alone and no treatment were independent risk factors for poor DSS, while surgery alone was an independent protective factor for better survival. Further investigations combining multiple fields of the gene modulation and molecular mechanism- are expected to elucidate better treatment strategies for cases of Ewing’s sarcoma of the bones and joints.

## References

[1] Uyeturk U, Helvaci K, Demirci A (2016). Clinical outcomes and prognostic factors of adult's Ewing sarcoma family of tumors: single center experience. Contemp Oncol (Pozn).

[2] Biswas B, Bakhshi S (2016). Management of Ewing sarcoma family of tumors: Current scenario and unmet need. World J Orthop.

[3] Delattre O, Zucman J, Melot T (1994). The Ewing family of tumors--a subgroup of small-round-cell tumors defined by specific chimeric transcripts. N Engl J Med.

[4] Esiashvili N, Goodman M, Marcus RB (2008). Changes in incidence and survival of Ewing sarcoma patients over the past 3 decades: Surveillance Epidemiology and End Results data. J Pediatr Hematol Oncol.

[5] Riggi N, Stamenkovic I (2007). The Biology of Ewing sarcoma. Cancer Lett.

[6] Rodriguez-Galindo C, Spunt SL, Pappo AS (2003). Treatment of Ewing sarcoma family of tumors: current status and outlook for the future. Med Pediatr Oncol.

[7] Teicher BA, Bagley RG, Rouleau C (2011). Characteristics of human Ewing/PNET sarcoma models. Ann Saudi Med.

[8] Saeter G, ESMo Guidelines Working Group (2007). Ewing's sarcoma of bone: ESMO clinical recommendations for diagnosis, treatment and follow-up. Ann Oncol.

[9] National Cancer Institute Surveillance, Epidemiology, and End Results Program. SEER*Stat Installation.

[10] National Cancer Institute (2002). Ewing Sarcoma Treatment (PDQ(r))-Health Professional Version.

[11] Delattre O, Zucman J, Plougastel B (1992). Gene fusion with an ETS DNA-binding domain caused by chromosome translocation in human tumours. Nature.

[12] Turc-Carel C, Aurias A, Mugneret F (1988). Chromosomes in Ewing's sarcoma. I. An evaluation of 85 cases of remarkable consistency of t(11;22)(q24;q12). Cancer Genet Cytogenet.

[B13] Yang Y, Wang H, Wei YY (2006). [Application of fluorescence in-situ hybridization and reverse transcription-polymerase chain reaction in molecular diagnosis of Ewing's sarcoma and primitive neuroectodermal tumor]. Zhonghua Bing Li Xue Za Zhi.

[14] Li S, Yang Q, Wang H (2016). Prognostic significance of serum lactate dehydrogenase levels in Ewing's sarcoma: A meta-analysis. Mol Clin Oncol.

[15] Arshi A, Sharim J, Park DY (2017). Prognostic determinants and treatment outcomes analysis of osteosarcoma and Ewing sarcoma of the spine. Spine J.

[16] Grevener K, Haveman LM, Ranft A (2016). Management and Outcome of Ewing Sarcoma of the Head and Neck. Pediatr Blood Cancer.

[17] Davenport JR, Vo KT, Goldsby R, West DC, DuBois SG (2016). Conditional Survival and Predictors of Late Death in Patients with Ewing Sarcoma. Pediatr Blood Cancer.

[18] Lee J, Hoang BH, Ziogas A, Zell JA (2010). Analysis of prognostic factors in Ewing sarcoma using a population-based cancer registry. Cancer.

[19] Ellis MA, Gerry DR, Neskey DM, Lentsch EJ (2017). Ewing Sarcoma of the Head and Neck. Ann Otol Rhinol Laryngol.

[20] Duchman KR, Gao Y, Miller BJ (2015). Prognostic factors for survival in patients with Ewing's sarcoma using the surveillance, epidemiology, and end results (SEER) program database. Cancer Epidemiol.

[21] Ahrens S, Hoffmann C, Jabar S (1999). Evaluation of prognostic factors in a tumor volume-adapted treatment strategy for localized Ewing sarcoma of bone: the CESS 86 experience. Cooperative Ewing Sarcoma Study. Med Pediatr Oncol.

[22] Karski EE, McIlvaine E, Segal MR (2016). Identification of Discrete Prognostic Groups in Ewing Sarcoma. Pediatr Blood Cancer.

[23] Rodriguez-Galindo C, Liu T, Krasin MJ (2007). Analysis of prognostic factors in ewing sarcoma family of tumors: review of St. Jude Children's Research Hospital studies. Cancer.

[24] Fizazi K, Dohollou N, Blay JY (1998). Ewing's family of tumors in adults: multivariate analysis of survival and long-term results of multimodality therapy in 182 patients. J Clin Oncol.

[25] Canter RJ (2016). Chemotherapy: Does Neoadjuvant or Adjuvant Therapy Improve Outcomes?. Surg Oncol Clin N Am.

[26] Oberlin O, Deley MC, Bui BN (2001). Prognostic factors in localized Ewing's tumours and peripheral neuroectodermal tumours: the third study of the French Society of Paediatric Oncology (EW88 study). Br J Cancer.

[27] Granowetter L, Womer R, Devidas M (2009). Dose-intensified compared with standard chemotherapy for nonmetastatic Ewing sarcoma family of tumors: a Children's Oncology Group Study. J Clin Oncol.

[28] DuBois SG, Krailo MD, Gebhardt MC (2015). Comparative evaluation of local control strategies in localized Ewing sarcoma of bone: a report from the Children's Oncology Group. Cancer.

[29] Bacci G, Longhi A, Briccoli A (2006). The role of surgical margins in treatment of Ewing's sarcoma family tumors: experience of a single institution with 512 patients treated with adjuvant and neoadjuvant chemotherapy. Int J Radiat Oncol Biol Phys.

[30] Bacci G, Longhi A, Ferrari S (2006). Prognostic factors in non-metastatic Ewing's sarcoma tumor of bone: an analysis of 579 patients treated at a single institution with adjuvant or neoadjuvant chemotherapy between 1972 and 1998. Acta Oncol.

[31] Jawad MU, Cheung MC, Min ES (2009). Ewing sarcoma demonstrates racial disparities in incidence-related and sex-related differences in outcome: an analysis of 1631 cases from the SEER database, 1973-2005. Cancer.

